# Promoting effective transitions: Primary school social–emotional competencies predict secondary school reading and numeracy achievement

**DOI:** 10.1111/bjep.12735

**Published:** 2025-01-17

**Authors:** Emma J. Carpendale, Melissa J. Green, Sonia L. J. White, Kate E. Williams, Stacy Tzoumakis, Oliver J. Watkeys, Felicity Harris, Kirstie O'Hare, Vaughan J. Carr, Kristin R. Laurens

**Affiliations:** ^1^ School of Psychology and Counselling Queensland University of Technology (QUT) Brisbane Queensland Australia; ^2^ Centre for Inclusive Education (C4IE) Queensland University of Technology (QUT) Brisbane Queensland Australia; ^3^ Centre for Child and Family Studies Queensland University of Technology (QUT) Brisbane Queensland Australia; ^4^ School of Clinical Medicine (Discipline of Psychiatry and Mental Health) University of New South Wales Sydney New South Wales Australia; ^5^ School of Early Childhood and Inclusive Education Queensland University of Technology (QUT) Brisbane Queensland Australia; ^6^ Australian Research Council Centre of Excellence for the Digital Child Canberra Australian Capital Territory Australia; ^7^ School of Education and Tertiary Access University of Sunshine Coast (USC) Sippy Downs Queensland Australia; ^8^ School of Criminology and Criminal Justice Griffith University Southport Queensland Australia; ^9^ Griffith Criminology Institute Griffith University Brisbane Queensland Australia; ^10^ Centre for Clinical Brain Sciences University of Edinburgh Edinburgh UK; ^11^ Department of Psychiatry Monash University Melbourne Victoria Australia

**Keywords:** academic achievement, Collaborative for Academic, Social and Emotional Learning, middle childhood, school success, social–emotional learning, student wellbeing

## Abstract

**Background:**

The transition from primary to secondary school presents a challenging developmental milestone which often marks a decline in academic performance. Social–emotional skills are recognized as fundamental to academic success but longitudinal research is needed to determine the extent of their association over this transition period.

**Aim:**

This study sought to determine the association between self‐reported social–emotional competencies of students in their final year of primary school (Year 6; age ~11 years) and reading and numeracy performance in their first year of secondary school (Year 7; age ~12 years).

**Sample:**

The study used a large Australian sample (*n =* 23,865), drawn from the New South Wales Child Development Study population cohort.

**Methods:**

The Middle Childhood Survey–Social–Emotional Learning assessment, administered during Year 6, comprises the five competencies defined by the Collaborative for Academic, Social and Emotional Learning (CASEL): Self‐Awareness, Self‐Management, Social Awareness, Relationship Skills and Responsible Decision‐Making. These data were linked with students' Year 7 reading and numeracy scores from the standardized National Assessment Program–Literacy and Numeracy measure. Associations were examined in multi‐level structural equation models which accounted for prior (Year 5) academic achievement and sociodemographic covariates. Multi‐group analyses explored invariance across girls and boys.

**Results:**

Self‐Awareness and Self‐Management demonstrated significant and meaningful positive relationships with reading and numeracy performance. Associations with reading were invariant by sex but boys demonstrated significantly stronger associations than girls on numeracy.

**Conclusion:**

Findings suggest that bolstering primary school students' intrapersonal social–emotional competencies may safeguard their academic achievement over the transition into secondary school.

## INTRODUCTION

A strong scientific and theoretical rationale supports fostering students' academic achievement by developing their school‐based social–emotional competencies. The capacity to follow directions, establish positive relationships in the classroom, maintain focus, regulate emotions and adjust to the school environment all support students' educational engagement and overall academic performance (Collie et al., 2019; Elias & Haynes, 2008; Zins et al., 2007). A meta‐analysis of 213 studies evaluating school‐based programs to develop students' social–emotional competencies observed sustained program effects on academic achievement, equating to an 11‐percentile gain (Durlak et al., 2011). However, the effect of programs delivered in middle childhood is under‐researched relative to programs delivered during preschool and early primary (Durlak et al., 2011; Jagers et al., 2015) or during secondary school (van de Sande et al., 2019). This presents a significant evidence gap as middle childhood typically incorporates a major, often challenging, school transition (The Centre for Adolescent Health, 2018).

In Australia, the transition from primary school (which incorporates 7 years of learning, between Kindergarten [age ~5 years] and Year 6 [age ~11 years]) to secondary school (comprising 6 years of learning, from Years 7 [age ~12 years] to 12 [age ~17 years]) involves changes to schooling environment, academic demands and social networks (Sniedze et al., 2021). This transition is characterized by a decline in academic achievement for many students (Carmichael, 2015; Hopwood et al., 2017). Deteriorating social–emotional well‐being may play a role in this academic decline. Waters et al. (2012) found that a third of their sample of Australian students self‐reported a ‘difficult’ primary to secondary school transition and experienced lower social–emotional well‐being than their peers up to a year later. Clarifying the relationship between social–emotional competencies and academic achievement following the secondary‐school transition may inform school‐based promotion of children's competencies.

The majority of formal social–emotional learning (SEL) curricula in schools internationally has been informed by the Collaborative for Academic, Social and Emotional Learning (CASEL, 2005, 2023) framework which defined five student social–emotional competencies (Self‐Awareness, Self‐Management, Social Awareness, Relationship Skills and Responsible Decision Making) described in Table 1.

**TABLE 1:** The five student social–emotional competencies defined by the Collaborative for Academic, Social and Emotional Learning.

Social–emotional competency	Definition
Self‐Awareness	Ability to understand one's own emotions, thoughts and values and how they influence behaviour across contexts
Self‐Management	Ability to manage one's emotions, thoughts and behaviours effectively in different situations and to achieve goals and aspirations
Social Awareness	Ability to understand the perspectives of and empathize with others, including those from diverse backgrounds, cultures and contexts
Relationship Skills	Ability to establish and maintain healthy and supportive relationships and to effectively navigate settings with diverse individuals and groups
Responsible Decision‐Making	Ability to make caring and constructive choices about personal behaviour and social interactions across diverse situations

A recent review of 12 meta‐analyses (Durlak et al., 2022) observed significant positive effects of primary and secondary school SEL programs on academic performance immediately post‐intervention (*d* = .27 to .46) (Durlak et al., 2011; Sklad et al., 2012; Wigelsworth et al., 2016) and at follow‐up of at least 6 months (*d* = .26 to .33) (Durlak et al., 2011; Sklad et al., 2012; Taylor et al., 2017). These small to moderate effects represent meaningful academic gain, equating to 11 to 18 percentiles for the average student (Durlak et al., 2022). Only a single meta‐analysis (Goldberg et al., 2019) did not observe significant effects of SEL interventions on academic achievement. In that analysis, only 8 (of 45) included studies focussed on academic outcomes (as opposed to the more commonly investigated outcomes of emotional and behavioural problems). Moreover, only a single study (of 8) had used standardized tests of academic achievement. Research in this space predominantly operationalises academic achievement as teacher‐based school grades; however, these measures are less objective than standardized assessments (McMillan, 2001).

Among studies that examined the association of social–emotional skills with academic achievement, few have explored effects across all five CASEL constructs. This may reflect the limited availability of validated self‐report measures aligned with these constructs, particularly for middle childhood (Denham, 2015). Ross and Tolan (2018) explored cross‐sectional associations between achievement (on a composite measure of school grades) and all five CASEL social–emotional competencies, using a self‐report measure developed among >1700 U.S. Year 5 students (age ~11 years old) and re‐administered to this cohort in Years 6 and 7. Cross‐sectional analyses were conducted in Years 5, 6 and 7 and found that four of CASEL's five competencies, barring Relationship Skills, were positively associated with school grades in Years 6 and 7 (Ross & Tolan, 2018) but only Self‐Awareness was associated with students' grades at the earliest (Year 5) assessment. This suggests that the relationship between competencies and academic achievement may vary by year level/stage. Research exploring these relationships at the time of transition from primary to secondary school may help optimize competency promotion efforts to prevent academic decline.

Notably few studies investigate associations of the social–emotional competencies with literacy and numeracy, with most investigations using composite measures of academic achievement (Barber & Olsen, 2004). Yet, divergent evidence demonstrates common versus specific associations between social–emotional skills and reading and numeracy. An international Survey of Social‐Emotional Skills that measured social–emotional competencies as the Big Five personality domains observed different associations between specific social and emotional skills and reading, mathematics and arts achievement (Organization for Economic Co‐operation and Development [OECD], 2021). Conversely, a large meta‐analysis of 40 studies of students from Kindergarten through Year 12 identified comparable magnitudes of effect for SEL on reading (Cohen's *d* = .25), mathematics (*d* = .26) and science (*d* = .19) (Corcoran et al., 2018). Systematic review of high‐quality school‐based well‐being programs (not limited to SEL) delivered in Australia revealed small to moderate impacts on general academic achievement (*g* = .26) and numeracy (*g* = .10) and literacy learning (*g* = .07) (Dix et al., 2020). Yet, other Australian‐based research found that students' subjective well‐being (composed of depression, anxiety and positive affect) in Year 8 (~age 13 years) positively predicted Year 9 numeracy scores but not Year 9 reading scores (Cárdenas et al., 2022). Further research to determine whether social–emotional competencies relate differently to reading and numeracy attainment might support tailoring of school‐based supports to students' specific learning needs.

Though associations between social–emotional skills and grades (in reading, mathematics and arts) are present for both girls and boys (OECD, 2021), there are consistent differences between girls' and boys' social–emotional development (OECD, 2021) and reading and numeracy performance (Thomas et al., 2022). Results on many standardized national and international tests (including the Australian National Assessment Program‐Literacy and Numeracy [NAPLAN] and the Program for International Student Assessment) indicate that boys face more challenges in reading than girls (Cobb‐Clark & Moschion, 2017; OECD, 2019; Thomas et al., 2022), whereas boys demonstrate an advantage over girls in numeracy (Leder & Forgasz, 2018; OECD, 2019; Thomas et al., 2022). Gender differences in NAPLAN scores are present already by the first assessment in Year 3 and tend to increase with progression through school (Thomas et al., 2022). DiPrete and Jennings (2012) found that differences in social–emotional skills between girls and boys in kindergarten explained 46% of girls' advantage in reading at Year 5 and reduced the gender gap favouring boys in numeracy at Year 5 by 28%. Therefore, social–emotional competencies may be implicated in gender gaps for reading and numeracy, warranting investigation of gender differences in the associations between social–emotional competencies and reading and numeracy attainment.

This study examined the longitudinal relationship between students' social–emotional competencies in the final year of primary school (Year 6) and standardized reading and numeracy attainment in the first year of secondary school (Year 7). The research sought to determine the individual relationship of each of the five competencies with attainment in each of numeracy and reading, as well as the extent to which these competencies explained shared or unique variance. Analyses accounted for previous academic performance (Year 5) and various sociodemographic covariates, and potential differences in associations for girls and boys were tested. It was hypothesized that higher scores on the five social–emotional competency domains would be positively associated with both reading and numeracy achievement in Year 7 (Hypothesis 1 [H1]). It was further hypothesized that these effects would hold when controlling for Year 5 performance and sociodemographic covariates (H2); and would be similar in magnitude for reading and numeracy attainment, and consistent for girls and boys (H3).

## METHOD

### Participants and procedures

Eligible participants (*n* = 23,865) for this study were drawn from the population cohort of 91,597 children represented in the third wave of multi‐agency record linkage conducted for the New South Wales‐Child Development Study (NSW‐CDS: Green et al., 2024). These participants comprised children in the cohort who completed the 2015 self‐report Middle Childhood Survey (MCS; Laurens et al., 2017) during Year 6 (age ~11 years) and had linked standardized reading and numeracy scores at Years 5 (age ~ 10 years: 2013–2015) and 7 (age ~12 years: 2015–2017) from the NAPLAN (supplied by the NSW Education Standards Authority, on behalf of the Australian Curriculum Assessment and Reporting Authority [ACARA]). Ethical approval for the linkage was provided by the NSW Population and Health Services, ACT Health and ACT Calvary Health HRECs (HREC/18/CIPHS/49), with relevant data custodian approvals. Probabilistic linkage of records in state‐level datasets was conducted using ChoiceMaker software (Choice Maker Technologies, Inc.) by the Centre for Health Record Linkage (https://www.cherel.org.au/; see Supplementary Method A for further detail), under a waiver of consent process consistent with the Australian National Health and Medical Research Council's (2007) National Statement of Ethical Conduct in Human Research. False linkage rate across all record sets in the Wave 3 linkage was estimated at .5%.

The MCS (Laurens et al., 2017) was administered to Year 6 students enrolled at 829 government and non‐government schools between July and September of 2015, representing 35.0% of primary schools in NSW in 2015. Schools were recruited to the study via email to the principal (leader), and the online survey was administered by teachers to students during class time. Following child or parent opt‐out, absences, or loss due to server failure, complete MCS data were available for 26,837 students (30.3%), who were representative of the NSW population of Year 6 students on a range of demographic indices (Laurens et al., 2017).

From these 26,837 students with complete MCS data, students with missing Year 7 (*n* = 1688) or 5 (*n* = 993) reading and numeracy scores, or covariate information (*n* = 53), were removed (Figure S1). Further, as this study used Year 7 school membership to account for multi‐level clustering of students in schools, data were also removed for students who attended schools that provided fewer than five students with complete MCS and NAPLAN records (238 students, 111 schools). The final sample for this study comprised 23,865 students, who attended one of 819 schools in Year 6 (MCS records) and one of 641 schools in Year 7 (NAPLAN records). Student and school demographic information for these 819 schools (Table S1) was obtained from the ACARA, with the representativeness of the study sample demonstrated by the close alignment of these data to the characteristics of all 2371 NSW schools with a Year 6 enrolment in 2015 (Dix et al., 2019). Two‐thirds of students' Year 6 schools were government schools (66.9%) and a majority were located in metropolitan areas (62.9%), with state‐representative rates of socio‐educational disadvantage (bottom quartile: 25.8%), Aboriginal and Torres Strait Islander students (9.0%) and students with a language background other than English (23.7%).

### Measures

#### Outcome variables: Reading and numeracy scores

The NAPLAN is a mandatory, nationwide, standardized assessment of foundational literacy and numeracy skills administered annually to students enrolled in Years 3, 5, 7 and 9 at all Australian government and non‐government schools (ACARA, 2023). The standardized reading and numeracy scores from Year 7 measured achievement in the students' first year of secondary school (for >99% of students in this study, these were scores from the assessment conducted in 2016). Records from the Year 5 assessment (for >99% of participants, these were scores from 2014) were used in this study to control for students' prior (primary school) academic achievement. NAPLAN standardized scores range between a theoretical minimum of zero and a maximum of 1000 across the 7 years of learning typically provided between Years 3 and 9. All year levels are scored on the same scale, with gains expected at each subsequent year level. For this study, NAPLAN reading and numeracy scores were rescaled (divided by 100) to align more closely with the variance of the predictor variables and covariates.

#### Predictor variables: Social–emotional competencies

The 20‐item self‐report Middle Childhood Survey–Social–Emotional Learning (MCS‐SEL; Carpendale et al., 2023a) assessment measured individual students' levels of the five social–emotional competencies: Self‐Awareness (4 items), Self‐Management (3 items), Social Awareness (5 items), Relationship Skills (5 items) and Responsible Decision‐Making (3 items). Items were rated on a three‐level response scale: not true (scored 0), somewhat true (1) and certainly true (2). Prior psychometric investigation in the full sample of 26,837 children with complete MCS data demonstrated satisfactory internal consistency for the five factors (McDonald's *ω* ranging from .653 to .764), theoretically coherent construct validity patterns and full measurement invariance across sex and language background subgroups (Carpendale et al., 2023a, 2023b). Latent variables representing each of the five competences were used in the analytical models.

#### Covariates

Year 5 NAPLAN scaled scores indexed each student's prior (primary school) attainment in reading and numeracy. Sociodemographic covariates were coded as binary indicators. Each child's sex was determined by consensus across NSW‐CDS linked records (girls: coded 0; boys: 1). Parental education was derived according to the maximum rating of either parent obtained from any of the four NAPLAN assessments available in NSW‐CDS linked records (Years 3, 5, 7 and 9). A dichotomous indicator of lower parental education (Year 12 or below: coded 1) was examined relative to higher parental education (education beyond Year 12 standard [i.e., tertiary education, including certificates, diplomas, degrees]: coded 0). A composite index of student disability at school entry was coded from the Australian Government Department of Education's Australian Early Development Census 2009, based on kindergarten teacher‐reported responses regarding whether the student requires special assistance or has an impairment which impacts their capacity to work in the classroom (Brinkman et al., 2014). These teacher reports were based on medical diagnosis/diagnoses or information provided by a parent, guardian, or Indigenous Cultural Consultant. A dichotomous variable was created to compare children with any learning, emotional, or behavioural impairment at school entry (coded 1) with children without a report of disability at school entry (0).

### Statistical analysis

#### Multi‐level structural equation modelling

Statistical analyses were conducted in Mplus version 8.0 (Muthén & Muthén, 1998). Multi‐level models that accounted for clustering of students (Level 1) within schools at Year 7 (Level 2) were required based on large school‐level intraclass correlations (Hox, 2002) for reading and numeracy obtained from initial null models (school membership explained 25.5% of the variance in students' Year 7 reading scores and 35.3% of the variance in Year 7 numeracy scores).

A series of multi‐level structural equation models (MSEM) were conducted separately for reading and numeracy outcomes. Mean‐ and variance‐adjusted weighted‐least squares estimation (WLSMV) was employed, being the recommended estimator for categorical indicators (indicators for the social–emotional competencies) and non‐normally distributed data (Lei, 2009). Model fits were evaluated via the comparative fit index (CFI), Tucker‐Lewis index (TLI), root mean square error of approximation (RMSEA) and standardized root mean square residual (SRMR). Good fit was characterized by values of CFI and TLI >.950, RMSEA <.050 and SRMR <.050 (Bentler & Bonett, 1980; Hu & Bentler, 1998, 1999).

Model 1 explored the bivariate associations between each of the five social–emotional competencies and reading and numeracy scores, separately for each competency, using simple MSEMs containing random intercepts and fixed slope parameter estimates. In each model, one competency factor (comprising between three to five MCS‐SEL items for each factor) was regressed on either the students' Year 7 reading or numeracy score. To control for Type I error across the 10 models, a Bonferroni‐adjusted alpha threshold (*p* < .027) that accounted for the high correlation between reading and numeracy scores (*r* = .730) was employed (https://www.quantitativeskills.com/sisa/calculations/bonfer.htm). The practical significance of effects was evaluated according to the proportion of variance in academic achievement accounted for in each model. *R* ^2^ values of <.02 were considered negligible, .03 to .12 weak, .13 to .26 moderate and above .27 substantial (Cohen, 1988), with the exploration of effects at Model 1 ceasing for *R* ^2^ values below .02. Effect sizes were interpreted via standardized *β* values, which denoted the standard deviations of increase in NAPLAN score associated with an increase of one standard deviation in the factor score for the social–emotional competency. These were then converted into the equivalent increases in percentile for the average student (i.e., students performing at the mean of reading/numeracy).

Bivariate analyses (two‐level random intercept models) confirmed each of the four covariates (sex, parental education, disability status and Year 5 academic performance) as significant predictors of reading and numeracy performance (*p* < .05; detailed in Table S2 and described in Supplementary Results A). Thus, to control for the effect of these covariates, Model 2 was specified as a simple MSEM including random intercepts and fixed slope parameter estimates for each social–emotional competency, including the four covariates as Level 1 predictors simultaneously with the competency in the regression on reading or on numeracy. As a supplementary analysis, these models were run as random intercepts and coefficients models allowing the association between competencies and academic achievement to vary across schools (Supplementary Results B).

While the primary analyses were focused on determining the association of each of the five social–emotional competencies with reading and numeracy performance separately, supplementary analyses determined whether the competencies accounted for unique variance in reading and numeracy outcomes when simultaneously considering the other competencies. Any competency demonstrating a practically significant association with academic performance was incorporated into a single MSEM for reading and another for numeracy and the difference in the *R* ^2^ value of this combined model relative to the individual models was compared. Models were then repeated controlling for the four covariates.

#### Multi‐group structural equation modelling

Multi‐group analyses were conducted to determine whether the associations between the various social–emotional competencies and academic achievement differed between girls and boys and, at the request of a reviewer, between children with higher (beyond Year 12) and lower (up to and including Year 12) parental education. These analyses freed the parameter estimates in Model 2 across groups (and removed the equivalent covariate). The known classes function recommended by Asparouhov and Muthén (2012) was used because the groups were within clusters (i.e., girls and boys within schools). As WLSMV is not available for this function, these models used robust maximum likelihood (MLR) estimation. Wald parameter constraint tests determined the significance of differences between parameter estimates of the coefficient effect of social–emotional competencies for girls and boys and higher and lower parental education groups.

## RESULTS

### Student demographic characteristics

Half of the 23,865 students in this sample were girls (*n* = 11,966; 50.1%), 11.1% (*n* = 2645) had parent(s) with an education level of Year 12 or below and 3.4% (*n* = 823) had a teacher‐reported disability at school entry (Table S3).

### Multi‐level structural equation modelling

#### Model 1: Bivariate associations

**FIGURE 1:**
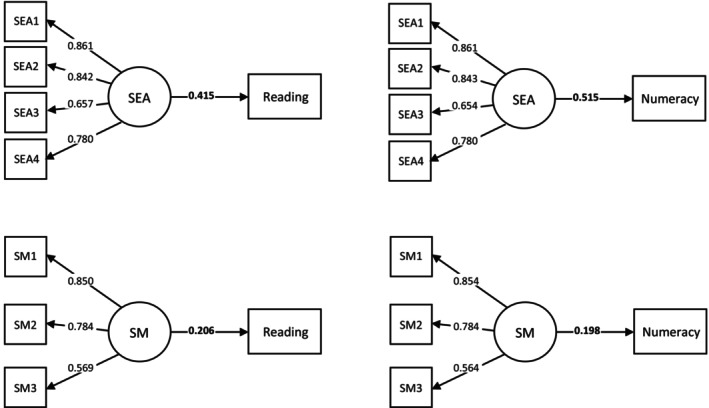
Standardized results of Two‐Level Structural Equation Models (Model 1) exploring the bivariate relationship between each social–emotional competency (Year 6) and reading and numeracy attainment (Year 7). Social Awareness, Relationship Skills and Responsible Decision‐Making did not demonstrate clinically meaningful associations with academic attainment so the results of these models are not reported. SEA = Self‐Awareness; SM = Self‐Management. Parameter estimates with dotted lines = non‐significant at *p* < .027 (corrected threshold), with solid lines = significant at *p* < .027 (corrected threshold), and parameter estimates in bold = significant at *p* < .001. Self‐Awareness and Reading: CFI = .998, TLI = .996, RMSEA = .035, SRMR = .013; Self‐Management and Reading: CFI = .987, TLI = .961, RMSEA = .068, SRMR = .022; Self‐Awareness and Numeracy: CFI = .998, TLI = .997, RMSEA = .031, SRMR = .014; Self‐Management and Numeracy: CFI = .994, TLI = .981, RMSEA = .048, SRMR = .019.

The MSEMs exploring bivariate associations between each social–emotional competency and students' Year 7 reading or numeracy score indicated statistically significant effects for all five competencies on both outcomes in this large population‐based sample. However, only the models for Self‐Awareness and Self‐Management met the designated *R* ^2^ threshold for practical significance (≥2.0%), explaining between 4.2% and 17.3% of the variance in reading and 3.9% and 26.5% of the variance in numeracy (Table 2 and Figure 1). The other three competencies accounted for <1% of the total variance in academic outcomes and their effects were not further explored.

**TABLE 2:** Proportion of variance in reading and numeracy attainment (Year 7) explained in the standardized Two‐level Structural Equation Models (Model 1 [bivariate association] and Model 2 [accounting for covariates]).

SEL competency	*R* ^2^			
	Reading		Numeracy	
	Model 1	Model 2	Model 1	Model 2
Self‐Awareness	.17***	.66***	.27***	.73***
Self‐Management	.04***	.65***	.04***	.71***
Social Awareness	<.01*		<.01*	
Relationship Skills	<.01**		<.01***	
Responsible Decision‐Making	<.01***		<.01	

*Note*: ***p < .001; **p < .010; *p < .027 (corrected threshold).

As detailed in the note to Figure 1, all four models demonstrated good model fit according to CFI, TLI, RSMEA and SRMR values (Bentler & Bonett, 1980; Hu & Bentler, 1998, 1999). The standardized regression coefficients (Table S4 provides the unstandardized coefficients) indicated that an increase of one standard deviation in Self‐Awareness was associated with an increase of .42 standard deviations (SD) in reading (equating to a 16‐percentile gain for an average student) and with .52 SD in numeracy (20‐percentile gain). Regarding Self‐Management effects, an increase of one SD equated to an increase of .21 SD in reading (8‐percentile gain) and .20 SD in numeracy (8‐percentile gain).

#### Model 2: Multivariate associations

**FIGURE 2:**
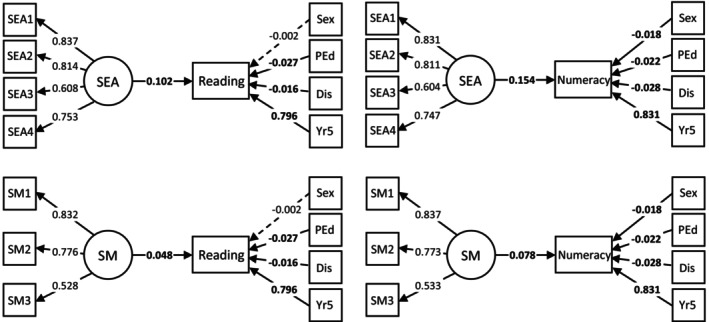
Standardized results of Two‐Level Structural Equation Models (Model 2) examining the relationship between each social–emotional competency (Year 6) and reading and numeracy attainment (Year 7), accounting for covariates. Dis = Disability; PEd = Parental Education; SEA = Self‐Awareness; SM = Self‐Management; Yr5 = Year 5 reading or numeracy score. Parameter estimates with dotted lines = non‐significant at *p* < .034 (corrected threshold), with solid lines = significant at *p* < .034 (corrected threshold), and parameter estimates in bold = significant at *p* < .001.

Figure 2 displays the standardized results for the two‐level random intercept MSEMs that controlled the effect of covariates, including prior academic achievement (Table S5 provides the unstandardized coefficients). *R* ^2^ values for Model 2 (Table 2) ranged from explaining 64.8% of the variance in reading scores (Self‐Management model) to 72.5% in numeracy scores (Self‐Awareness model). Irrespective of the strong relationship of prior (Year 5) academic performance with later reading and numeracy (Year 7), Self‐Awareness and Self‐Management remained significantly and positively associated with reading and of numeracy scores in the context of the four covariates. Though Year 5 academic achievement showed the strongest association with Year 7 achievement scores, social–emotional competencies were typically the next strongest association. Low parental education and disability status were negatively associated with reading and numeracy. Sex was a significant predictor only in the numeracy models, with boys demonstrating significantly lower numeracy scores than girls in the context of the other variables. Standardized coefficients indicate an increase of one SD in social–emotional competencies was associated with an increase in NAPLAN score, ranging from increases of .05 SD in reading (2‐percentile gain: Self‐Management model) to .15 SD in numeracy (6‐percentile gain: Self‐Awareness model).

In random intercept and coefficient models, the association between Self‐Awareness and academic achievement varied significantly across schools (see Supplementary Results B for results and additional details). Exploratory investigations suggest that the association between Self‐Awareness and numeracy is stronger among schools with higher average numeracy scores.

#### Supplementary analyses of unique versus shared variance

The supplementary multivariable MSEMs that included Self‐Awareness and Self‐Management in a single model per academic outcome indicated that each competency remained significantly associated with reading and numeracy (Tables S6 and S7). Nonetheless, the two competencies explained shared variance in students' academic performance, as indicated by the increase of <1% in *R* ^2^ for the combined models relative to *R* ^2^ values from the bivariate Self‐Awareness models (Table 2).

### Multi‐group structural equation modelling (sex invariance)

Table 3 displays the multi‐group MSEM results for girls and boys separately, and the Wald parameter constraint test comparing the associations between competencies and outcomes across girls and boys. Self‐Awareness was a significant positive predictor of numeracy for both girls (*β* = .17, *p* < .001) and boys (*β* = .18, *p* < .001), but boys had a significantly stronger association according to the Wald test (Wald test = 7.49, *p* = .006). Similarly, the association between Self‐Management and numeracy was significantly stronger for boys (*β* = .08, *p* < .001) than for girls (*β* = .07, *p* < .001), according to the Wald test (Wald test = 4.79, *p* = .029). Results related to reading achievement were invariant by sex. No significant Wald test comparisons were observed when comparing effects between children with higher (beyond Year 12) and lower (up to Year 12) parental education (Table S8).

**TABLE 3:** Results of Multi‐Group Two‐Level Structural Equation Models examining the relationship between each social‐emotional competency (Year 6) and reading and numeracy attainment (Year 7) across girls and boys and accounting for covariates.

Parameter	Reading								Numeracy							
	Girls				Boys				Girls				Boys			
	*β*	*B*	*SE*	*t*	*β*	*B*	*SE*	*t*	*β*	*B*	*SE*	*t*	*β*	*B*	*SE*	*t*
Self‐Awareness																
SEA Coefficient	.105	.022	(.00)	14.22***	.116	.025	(.00)	14.15***	.166	.033	(.00)	20.31***	.177	.038	(.00)	21.66***
Wald test (*df*)^a^	2.25 (1)								7.49 (1)**							
PEd Coefficient	−.025	−.053	(.01)	−4.28***	−.029	−.060	(.01)	−4.60***	−.027	−.053	(.01)	−5.22***	−.017	−.037	(.01)	−2.87**
Dis Coefficient	−.015	−.067	(.03)	−2.27	−.015	−.046	(.02)	−2.15	−.027	−.118	(.02)	−5.21***	−.026	−.084	(.02)	−4.91***
Yr5 Coefficient	.777	.634	(.01)	106.78***	.788	.626	(.01)	104.60***	.788	.706	(.01)	97.17***	.824	.719	(.01)	99.94***
Self‐Management																
SM Coefficient	.043	.010	(.02)	5.25***	.056	.014	(.00)	7.81***	.069	.015	(.00)	9.37***	.084	.021	(.00)	11.69***
Wald test (*df*)^a^	2.05 (1)								4.79 (1)*							
Ped Coefficient	−.024	−.051	(.01)	−4.13***	−.027	−.059	(.01)	−4.54***	−.026	−.053	(.01)	−5.06***	−.016	−.036	(.01)	−2.76**
Dis Coefficient	−.014	−.067	(.03)	−2.26	−.015	−.049	(.02)	−2.30	−.025	−.115	(.02)	−4.87***	−.026	−.086	(.02)	−5.04***
Yr5 Coefficient	.788	.657	(.01)	117.48***	.800	.651	(.01)	114.02***	.807	.753	(.01)	108.98***	.843	.769	(.01)	112.86***

*Note*: ***p < .001; **p < .010; *p < .034 (corrected threshold).

Abbreviations: *df*, degrees of freedom; Dis, Disability; PEd, Parental Education; SEA, Self‐Awareness; SM, Self‐Management; Yr5, Year 5 reading or numeracy score.

^a^ Wald test of the significance of the difference between the competency coefficient parameters of girls and boys.

## DISCUSSION

This study of a representative sample of Australian students found that two of the five CASEL social–emotional competencies measured in Year 6 were moderately associated with academic achievement a year later, following transition to secondary school. In particular, Self‐Awareness and Self‐Management were significantly associated with both Year 7 reading and numeracy (H1), even when controlling for prior achievement in Year 5 and sociodemographic covariates (H2). Effects were consistent for girls and boys on reading, but competencies were more strongly related to numeracy for boys than girls (H3). These findings endorse the fostering of students' intrapersonal social–emotional skills and suggest specific social–emotional targets for classroom initiatives and school‐based programs (Beatson et al., 2023) to support students' learning across the challenging transition from primary to secondary school.

Self‐Awareness and Self‐Management explained a practically significant proportion of variance in reading and numeracy attainment, with bivariate models explaining between 4% and 27% of variation. One standard deviation increase in skills was associated with an increase equivalent to between 8‐ to 20‐percentiles in attainment for an average student at the bivariate level and between 2‐ to 6‐percentiles when accounting for prior academic achievement and covariates. The size of these standardized effects when accounting for prior achievement, sex, disability and parental education (*β* ranging from .05 to .15) closely align with findings of the OECD (2021) Survey of Social and Emotional Skills. This survey explored the association of social–emotional skills and reading and mathematics achievement when accounting for gender, socioeconomic status and cognitive ability, and observed effects ranging up to .08 for 10‐year‐olds and up to .15 for 15‐year‐olds.

There is a strong theoretical and empirical base underpinning these observed associations. Self‐Awareness (also conceptualized as self‐confidence or academic self‐efficacy) affords students confidence in their capacity to accomplish academic tasks and supports them in their endeavours to participate, engage, face challenges and ask for help (Denham, Bassett, Mincic, et al., 2012; Denham & Brown, 2010; Ferla et al., 2009). Bandura's (1978) theory of reciprocal determinism denotes that learners with higher self‐efficacy are more likely to set higher goals for themselves and persevere in the face of challenges, with this determination then paving the way for academic success, which in turn fosters stronger academic self‐efficacy (Williams & Williams, 2010). Accumulating evidence supports this bidirectional association between Self‐Awareness and academic achievement (Denham & Brown, 2010; Zafiropoulou et al., 2007). Across the transition to secondary school, stronger self‐esteem and academic self‐efficacy could mitigate against the decline in perceived control over learning commonly observed across this transition period and encourage perseverance despite these challenges (Evans et al., 2018). With respect to Self‐Management, children's capacity for attentional, emotional and behavioural regulation supports positive classroom engagement and helps students to focus on their learning (McClelland et al., 2006; McClelland & Wanless, 2012; Trentacosta & Izard, 2007). The Lattice Model of Reading positions self‐regulation and motivation skills as integral aspects of reading development, as these skills support students to focus their attention, follow directions and persist in their learning (Connor, 2016). With the new academic demands of secondary school, children with stronger skills in sustaining attention, persisting with difficult tasks and employing effective strategies to achieve goals will be better equipped to adapt to these changes (Xia et al., 2016). Previous research with CASEL competencies partly aligns with our results, as Ross and Tolan (2018) found that Self‐Awareness and Self‐Management were the two strongest predictors of academic achievement in middle school, specifically Years 6 and 7. The present study contributes to the growing evidence base positioning learning‐related, intrapersonal social–emotional skills as prominent contributors to academic achievement (McClelland et al., 2000; Oberle et al., 2014; Soland & Kuhfeld, 2022).

Effects in the present study were common across reading and numeracy rather than domain‐specific, aligning broadly with the findings of prior meta‐analyses (Corcoran et al., 2018; Dix et al., 2020). This suggests that delivery of universal teaching to bolster Self‐Awareness and Self‐Management skills may offer an effective means of fostering students' academic functioning across different areas of the curriculum. Notably, supplementary analyses indicated that Self‐Awareness and Self‐Management explain overlapping variance in academic attainment, such that shared aspects of these competencies drive these associations with reading and numeracy. Potentially, these shared aspects might be attributable to improved executive functioning (Zelazo & Carlson, 2020), reduction in mental health difficulties (Denham, Bassett, Thayer, et al., 2012; Panayiotou et al., 2019), improved quality of student‐teacher relationships (Hernández et al., 2017; Valiente et al., 2008) and/or school liking (Valiente et al., 2007).

In contrast to prior research demonstrating associations between academic attainment and Relationship Skills, Social Awareness and Responsible Decision‐Making (Elias & Haynes, 2008; Ross & Tolan, 2018), the present study did not identify practically meaningful associations. While Ross and Tolan (2018) found cross‐sectional associations between these competencies and school grades, the longitudinal nature of the current study means that potential declines in social–emotional well‐being following the transition to secondary school may have attenuated the effects of these three competencies on academic achievement. Consistent with the present findings, Oberle et al. (2014) found that self‐reported social responsibility goals in Year 6 were not associated with Year 7 reading performance for girls, nor with numeracy performance for girls or boys. However, teacher‐reported social skills were significant predictors of these outcomes (Oberle et al., 2014). Future research should focus on exploring the temporal relationship of associations and informant‐specific effects. Nonetheless, the importance of these three competencies for a variety of other student outcomes, particularly peer relationships, social well‐being and curtailing of problem behaviours (Domitrovich et al., 2017; Guerra & Bradshaw, 2008; Milledge et al., 2019; Waschbusch et al., 2007), means they should not be discounted as important targets (alongside academic achievement) for school‐based SEL.

With respect to sex effects, there was an advantage for girls in reading and for boys in numeracy at the bivariate level; however, these effects attenuated for the reading models and reversed for the numeracy models once other covariates were considered. This may be because the multivariable models (Model 2) accounted for Year 5 achievement, where a gender gap is also present (Thomas et al., 2022). The multi‐group MSEM analyses indicated consistent effects of competencies on reading for girls and boys, aligning with previous research using teacher‐reported social–emotional skills (Oberle et al., 2014). However, the effect of Self‐Awareness and Self‐Management on numeracy was stronger for boys than girls. Thomas et al. (2022) and Leder and Forgasz (2018) have speculated that the advantage of boys in numeracy performance is propagated by societal expectations and stereotypes that boys outperform girls in mathematics. Therefore, societal expectations might augment the role of intrapersonal competencies in supporting boys' numeracy learning relative to girls, the latter for whom lower math‐confidence is often reified by these stereotypes. Nonetheless, this study demonstrates that social–emotional competencies are significantly associated with academic achievement for both girls and boys, thus endorsing the universal delivery of formal teaching of social–emotional competencies in schools.

There are several limitations of this study. First, this study was restricted to measuring social–emotional competencies in Year 6 and did not capture potential changes to social–emotional functioning in Year 7, following the transition to secondary school. Consistent, repeated assessments are needed to track students' social–emotional skills and enhance understanding of their associations with academic achievement concurrently and longitudinally. Further, this study was not able to control for the potential effects of any SEL delivered in Year 7, as this was not measured. As a brief (20‐item) measure suitable for population administration, the MCS‐SEL instrument (Carpendale et al., 2023a) provides only limited coverage of the skills reflected in each of the five CASEL competencies and this might explain the negligible effects observed for three of the five competencies. Conversely, findings for Self‐Awareness might be overestimated, as the scale was predominantly oriented towards the learning‐related aspects of Self‐Awareness (e.g., “*I easily learn my schoolwork*”). The study sample excluded students with missing NAPLAN data (10%), with the reasons for this missing data (e.g., illness, absence, withdrawal) being unavailable; nonetheless, the sample closely resembled the NSW population of Year 6 students and schools. While the NAPLAN is a standardized assessment that is more objective than teacher‐rated grades (McMillan, 2001), the invariance of the NAPLAN measure across diverse populations has not been confirmed. Similarly, research determining whether social–emotional competencies support academic performance to the same degree among students of diverse cultural and socioeconomic backgrounds is needed, as this may indicate the need for tailored provision of school‐based support. Though this study includes the successive timing of measures (Years 5, 6 and 7), future research should seek to explore whether functioning across the CASEL constructs assessed earlier in development also predicts secondary school academic achievement or whether these effects are specific to proximal social–emotional functioning. Future research exploring mediators and moderators of these associations would improve understanding of the mechanisms underlying the complex relationship between social–emotional competencies and academic achievement.

## CONCLUSION

This large‐scale, longitudinal investigation demonstrates the association of students' social–emotional functioning on Self‐Awareness and Self‐Management in Year 6 with Year 7 reading and numeracy achievement for girls and boys across the challenging transition from primary to secondary school. These findings endorse the national movement towards placing school‐based promotion of social–emotional well‐being on an equal footing to academic learning (Productivity Commission, 2020, 2023). Focusing on the “whole child” and universally supporting development of social–emotional competencies during primary school, particularly intrapersonal skills, may not only foster stronger social–emotional well‐being but also provide students with resources conducive to stronger learning into secondary school.

## AUTHOR CONTRIBUTIONS

**Emma J. Carpendale:** Conceptualization; investigation; writing – original draft; methodology; visualization. **Melissa J. Green:** Conceptualization; funding acquisition; writing – review and editing; project administration; supervision; data curation; methodology. **Sonia L. J. White:** Conceptualization; writing – review and editing; supervision; methodology. **Kate E. Williams:** Conceptualization; writing – review and editing; supervision; methodology. **Stacy Tzoumakis:** Funding acquisition; writing – review and editing; data curation. **Oliver J. Watkeys:** Writing – review and editing; data curation. **Felicity Harris:** Writing – review and editing; project administration; data curation. **Kirstie O'Hare:** Writing – review and editing; data curation. **Vaughan J. Carr:** Writing – review and editing; data curation. **Kristin R. Laurens:** Conceptualization; investigation; writing – review and editing; project administration; funding acquisition; supervision; data curation; methodology.

## FUNDING INFORMATION

This research was conducted by the University of New South Wales with financial support from National Health and Medical Research Council Project Grants (APP1058652 and APP1148055), an Australian Research Council Future Fellowship (FT170100294 awarded to K.R.L.) and Discovery Early Career Researcher Award (DE210100113 awarded to S.T.), and a Department of Health and Aged Care Medical Research Future Fund Million Minds Mental Health Grant (APP2006436). E.J.C. was supported by an Australian Government Research Training Program Stipend scholarship and a Queensland University of Technology Faculty of Health Excellence Top‐Up scholarship.

## CONFLICT OF INTEREST STATEMENT

The authors have no conflicts of interest to disclose.

## Supporting information


**Data S1.** Supporting information.

## Data Availability

Privacy legislation and ethical restrictions placed on the use of de‐identified multi‐agency linked government data limit access to the study data, which cannot be made publicly available. Collaborative research activities may be possible depending on scope and resources.
