# Supplementary material for: Promoting effective transitions: Primary school social–emotional competencies predict secondary school reading and numeracy achievement
Source: Br J Educ Psychol. 2025 Jan 17;95(2):496–512. doi: 10.1111/bjep.12735 (PMC12068035; doi:10.1111/bjep.12735)
Supplement: Supplementary file 1 — Data S1. Supporting information. [file BJEP-95-496-s001.docx]

**Promoting Effective Transitions: Primary School Social-Emotional Competencies**

**Predict Secondary School Reading and Numeracy Achievement**

Emma J. Carpendale, Melissa J. Green, Sonia L. J. White, Kate E. Williams, Stacy Tzoumakis,

Oliver J. Watkeys, Felicity Harris, Kirstie O’Hare, Vaughan J. Carr, and Kristin R. Laurens

***Supplementary Materials***

**Contents:**

*Supplementary Method A:* Description of probabilistic record linkage conducted by the *Centre for Health Record Linkage* using *ChoiceMaker* software (Choice Maker Technologies Inc.). 2

*Supplementary Figure S1:* Flow diagram displaying the process of deriving the final sample of 23,865 students for this study. 3

*Supplementary Table S1:* Sample characteristics according to Year 6 school, relative to the characteristics of all NSW schools with a Year 6 enrolment in 2015 (from Dix et al., 2019)*.* 4

*Supplementary Results A:* Summary of bivariate associations between each covariate and reading and numeracy attainment (Year 7) 4

*Supplementary Table S2:* Results of Two-Level Structural Equation Models (random intercept model) exploring the bivariate association between each covariate and reading and numeracy attainment (Year 7). 4

*Supplementary Table S3:* Sample characteristics according to predictors, outcomes, and covariates 5

*Supplementary Table S4:* Results of Two-Level Structural Equation Models (Model 1) exploring the bivariate relationship between each social-emotional competency (Year 6) and reading and numeracy attainment (Year 7) 5

*Supplementary Table S5:* Results of the Two-Level Structural Equation Model (Model 2) exploring the relationship between each social-emotional competency (Year 6) and reading and numeracy attainment (Year 7), accounting for covariates. 6

*Supplementary Results B:* Summary of random intercept and coefficients model results. 7

*Supplementary Table S6:* Results of the Two-Level Structural Equation Model (Model 1) exploring the relationship between both Self-Awareness and Self-Management (Year 6) and reading and numeracy attainment (Year 7). 8

*Supplementary Table S7:* Results of the Two-Level Structural Equation Model (Model 2) exploring the relationship between both Self-Awareness and Self-Management (Year 6) and reading and numeracy attainment (Year 7), and accounting for covariates. 8

*Supplementary Table S8:* Unstandardized results of Multi-Group Two-Level Structural Equation Models examining the relationship between each social-emotional competency (Year 6) and reading and numeracy attainment (Year 7) according to parents’ level of education, accounting for covariates. 9

**Supplementary Method A.** Description of probabilistic record linkage conducted by the *Centre for Health Record Linkage* using *ChoiceMaker* software (Choice Maker Technologies Inc.).

This method used machine learning to assign linkage weights that represented the probability of the records matching on personal identifiers (e.g., first name, surname, date of birth, residential address and postcode), accommodating missing identifiers in some records. Possible matches were sorted into matches, potential matches (which were reviewed by staff), and non-matches based on user-defined thresholds (adjusted to ensure false links were kept to a minimum), and any record matches with a probability higher than the ‘match threshold’ were declared a ‘match’.

**Supplementary Figure S1.** Flow diagram displaying the process of deriving the final sample of 23,865 students for this study.

Middle Childhood Survey (MCS) administered

(*n* = 27,792 students;

829 schools)

Complete MCS social-emotional competency data

(*n* = 26,837 students)

Complete MCS social-emotional competency data and NAPLAN scores

(*n* =24,156 students)

Complete MCS social-emotional competency data, NAPLAN scores, and covariates

(*n* =24,103 students)

Complete data available for multilevel modelling

(*n* = 23,865 students;

819 Year 6 schools;

641 Year 7 schools)

Students without complete MCS data

(*n* = 955 students)

Students with missing NAPLAN data in Year 7 or Year 5

(*n* = 2,681 unique students):

- Year 7 reading (*n* = 1,389)

- Year 7 numeracy (*n* = 1,541)

- Year 5 reading (*n* = 1,226)

- Year 5 numeracy (*n* = 1,277)

Students with missing parental education data

(*n* = 53 students)

Students with missing Year 7 school code and/or students attending a Year 7 school that has fewer than 5 students in this sample

(*n* = 238 students; 111 Year 7 schools)

NSW Child Development Study cohort at Wave 3 Linkage

(*n* = 91,597 students)

Students who did not participate in Middle Childhood Survey

(*n* = 63,805 students)

**Table S1.** Sample characteristics according to Year 6 school, relative to the characteristics of all 2,371 NSW schools with a Year 6 enrolment in 2015 (from Dix et al., 2019).

| **Variables** | Sample *%* (*n*) | NSW schools % |
| --- | --- | --- |
| School sector |  |  |
| Government | 66.9 (548) | 67.9 |
| Non-Government | 33.1 (271) | 32.1 |
| Geographical Location |  |  |
| Metropolitan | 62.9 (515) | 59.9 |
| Provincial/Remote/Very Remote | 37.1 (304) | 40.1 |
| Socio-educational Advantage (ICSEA)^a^ |  |  |
| Quartile 1 (most disadvantaged) | 25.8 (211) | 28.8 |
| Quartile 4 (most advantaged) | 25.2 (206) | 23.5 |
|  | *Mean (SD)* | *Mean (SD)* |
| Percentage of students at the school of Aboriginal and Torres Strait Islander background | 9.0 (11.9) | 9.1 (13.7) |
| Percentage of students at the school who spoke a language other than English at home | 23.7 (27.3) | 23.3 (27.3) |

***Note.*** *n* = number of participants; *SD* = standard deviation; **^a^** Quartile position of school’s socio-educational advantage score according to the nationally derived *Index of Community Socio-Educational Advantage* (ICSEA).

Dix, K. L., Green, M. J., Tzoumakis, S., Dean, K., Harris, F., Carr, V. J., & Laurens, K. R. (2019). The Survey of School Promotion of Emotional and Social Health (SSPESH): a brief measure of the implementation of whole-school mental health promotion. *School Mental Health, 11*(2), 294-308. <https://doi.org/10.1007/s12310-018-9280-5>

**Supplementary Results A:** Summary of bivariate associations between each covariate and reading and numeracy attainment (Year 7).

Two-level random intercept models found significant bivariate associations between all four covariates (sex, parental education, disability status, and Year 5 NAPLAN scores) and Year 7 reading and numeracy scores (Supplementary Table S2). Numeracy and reading outcomes were significantly lower for students with parental education levels of up to or below Year 12 equivalent, and for students with a disability reported at school entry. Boys demonstrated significantly lower reading scores than girls, but significantly higher numeracy scores than girls. Year 5 academic achievement scores were strongly positively associated with Year 7 achievement scores.

**Table S2.** Results of Two-Level Structural Equation Models (random intercept model) exploring the bivariate association between each covariate and reading and numeracy attainment (Year 7).

|  | **Reading** | | | | | **Numeracy** | | | | |
| --- | --- | --- | --- | --- | --- | --- | --- | --- | --- | --- |
| **Covariate** | **β** | **B** | **SE** | ***t*** | **R^2^** | **β** | **B** | **SE** | ***t*** | **R^2^** |
| Sex (male) | -0.047 | -0.058 | (0.01) | -7.13*** | .002*** | 0.060 | 0.074 | (0.01) | 8.51*** | .004*** |
| Parental Education (<Year 12) | -0.111 | -0.217 | (0.01) | -16.99*** | .012*** | -0.103 | -0.201 | (0.01) | -16.52*** | .011*** |
| Disability (Reported) | -0.094 | -0.318 | (0.02) | -16.29*** | .009*** | -0.113 | -0.380 | (0.02) | -18.89*** | .013*** |
| Year 5 NAPLAN score | 0.803 | 0.663 | (0.00) | 210.22*** | .644*** | 0.836 | 0.773 | (0.01) | 251.23*** | .699*** |

*Note.* *** *p*< .001; ** *p*< .01; * *p*< .050.

**Table S3.** Sample characteristics according to predictors, outcomes, and covariates.

| **Variables** | ***n* (%)** |  |  |  |
| --- | --- | --- | --- | --- |
| Total Sample | 23,865 |  |  |  |
| Sex |  |  |  |  |
| Boys | 11,899 (49.9) |  |  |  |
| Girls | 11,966 (50.1) |  |  |  |
| Parental Education Level (highest of either parent) |  |  |  |  |
| Up to Year 12 | 2,645 (11.1) |  |  |  |
| Beyond Year 12 | 21,220 (88.9) |  |  |  |
| AEDC Disability (Learning, Emotional, and/ or Behavioural) |  |  |  |  |
| Reported | 823 (3.4) |  |  |  |
| Not reported | 23,042 (96.6) |  |  |  |
|  | **Summed Scores** | | **Factor Scores** | |
| **Social-Emotional Competency Scores** | ***Mean (SD)*** | ***Range*** | ***Mean (SD)*** | ***Range*** |
| *Self-Awareness* | 5.65 (1.64) | 0 to 8 | -0.00 (0.82) | -3.02 to 1.47 |
| *Self-Management* | 4.74 (1.42) | 0 to 6 | -0.04 (0.76) | -2.81 to 1.24 |
| *Social Awareness* | 7.86 (1.88) | 0 to 10 | -0.03 (.084) | -3.58 to 1.62 |
| *Relationship Skills* | 7.30 (2.16) | 0 to 10 | -0.03 (0.83) | -3.24 to 1.53 |
| *Responsible Decision-Making* | 3.92 (1.65) | 0 to 6 | -0.03 (0.81) | -2.44 to 1.33 |
|  | **Scaled Scores** | | **Rescaled Scores** | |
| **NAPLAN Scores** | ***Mean (SD)*** | ***Range*** | ***Mean (SD)*** | ***Range*** |
| Year 5 NAPLAN scores |  |  |  |  |
| Reading | 507.90 (81.61) | 86.90 to 811.40 | 5.08 (0.81) | 0.87 to 8.11 |
| Numeracy | 496.19 (74.39) | 122.00 to 814.30 | 4.96 (0.74) | 1.22 to 8.14 |
| Year 7 NAPLAN scores |  |  |  |  |
| Reading | 545.42 (70.11) | 113.20 to 909.40 | 5.45 (0.70) | 1.13 to 9.09 |
| Numeracy | 555.19 (74.82) | 219.20 to 875.40 | 5.55 (0.75) | 2.19 to 8.75 |

*Note. n* = number of participants; *SD* = standard deviation.

**Table S4.** Results of Two-Level Structural Equation Models (Model 1) exploring the bivariate relationship between each social-emotional competency (Year 6) and reading and numeracy attainment (Year 7).

|  | **Reading** | | | | **Numeracy** | | | |
| --- | --- | --- | --- | --- | --- | --- | --- | --- |
| **Parameter** | **β** | **B** | ***SE*** | ***t*** | **β** | **B** | ***SE*** | ***t*** |
| *Self-Awareness* |  |  |  |  |  |  |  |  |
| SEA1 Item Loading | 0.861 | 1.000 |  |  | 0.861 | 1.000 |  |  |
| SEA2 Item Loading | 0.842 | 0.924 |  |  | 0.843 | 0.929 |  |  |
| SEA3 Item Loading | 0.657 | 0.516 |  |  | 0.654 | 0.512 |  |  |
| SEA4 Item Loading | 0.780 | 0.739 |  |  | 0.780 | 0.739 |  |  |
| SEA Coefficient | 0.415 | 0.152 | (0.003) | 44.41*** | 0.515 | 0.187 | (0.004) | 46.55*** |
| *Self-Management* |  |  |  |  |  |  |  |  |
| SM1 Item Loading | 0.850 | 1.000 |  |  | 0.854 | 1.000 |  |  |
| SM2 Item Loading | 0.784 | 0.785 |  |  | 0.784 | 0.770 |  |  |
| SM3 Item Loading | 0.569 | 0.429 |  |  | 0.564 | 0.416 |  |  |
| SM Coefficient | 0.206 | 0.079 | (0.004) | 19.59*** | 0.198 | 0.074 | (0.004) | 19.26*** |

*Note.* *** *p*< .001; ** *p*< .01; * *p*< .027 (corrected threshold). SEA = Self-Awareness; SM = Self-Management.

**Table S5.** Results of the Two-Level Structural Equation Model (Model 2) exploring the relationship between each social-emotional competency (Year 6) and reading and numeracy attainment (Year 7), accounting for covariates.

|  | **Reading** | | | | **Numeracy** | | | |
| --- | --- | --- | --- | --- | --- | --- | --- | --- |
| **Parameter** | **β** | **B** | ***SE*** | ***t*** | **β** | **B** | ***SE*** | ***t*** |
| *Self-Awareness* |  |  |  |  |  |  |  |  |
| SEA1 Item Loading | 0.837 | 1.000 |  |  | 0.831 | 1.000 |  |  |
| SEA2 Item Loading | 0.814 | 0.915 |  |  | 0.811 | 0.926 |  |  |
| SEA3 Item Loading | 0.608 | 0.500 |  |  | 0.604 | 0.506 |  |  |
| SEA4 Item Loading | 0.753 | 0.747 |  |  | 0.747 | 0.752 |  |  |
| SEA Coefficient | 0.102 | 0.045 | (0.002) | 21.41*** | 0.154 | 0.071 | (0.003) | 24.32*** |
| Sex Coefficient | -0.002 | -0.002 | (0.005) | -0.22 | -0.018 | -0.025 | (0.005) | -4.80*** |
| PEd Coefficient | -0.027 | -0.058 | (0.008) | -7.01*** | -0.022 | -0.048 | (0.008) | -6.09*** |
| Dis Coefficient | -0.016 | -0.061 | (0.012) | -4.91*** | -0.028 | -0.105 | (0.014) | -7.58*** |
| Yr5 Coefficient | 0.796 | 0.659 | (0.003) | 204.90*** | 0.831 | 0.770 | (0.003) | 242.22*** |
| *Self-Management* |  |  |  |  |  |  |  |  |
| SM1 Item Loading | 0.832 | 1.000 |  |  | 0.837 | 1.000 |  |  |
| SM2 Item Loading | 0.776 | 0.822 |  |  | 0.773 | 0.795 |  |  |
| SM3 Item Loading | 0.528 | 0.415 |  |  | 0.533 | 0.411 |  |  |
| SM Coefficient | 0.048 | 0.022 | (0.002) | 9.16*** | 0.078 | 0.035 | (0.002) | 14.62*** |
| Sex Coefficient | -0.002 | -0.002 | (0.005) | -0.42 | -0.018 | -0.025 | (0.005) | -4.81*** |
| PEd Coefficient | -0.027 | -0.058 | (0.008) | -7.01*** | -0.022 | -0.048 | (0.008) | -6.09*** |
| Dis Coefficient | -0.016 | -0.061 | (0.012) | -4.91** | -0.028 | -0.105 | (0.014) | -7.58*** |
| Yr5 Coefficient | 0.796 | 0.659 | (0.003) | 204.90*** | 0.831 | 0.770 | (0.003) | 242.22*** |

*Note.* *** *p*< .001; ** *p*< .01; * *p*< .034 (Bonferroni-adjusted significance threshold for 4 models and 0.730 correlation between reading and numeracy). SEA = Self-Awareness; SM = Self-Management; PEd = Parental Education; Dis = Disability; Yr5 = Year 5 NAPLAN score.

**Supplementary Results B:** Summary of random intercept and coefficients model results.

Model 2 Multi-level Structural Equation Modelling analyses (accounting for covariates) were repeated as random intercept and coefficients models that tested between-school differences in the relationships between social-emotional competencies (Self-Awareness and Self-Management) and academic achievement (reading and numeracy). Accordingly, the impact of social-emotional competencies on academic achievement was free to vary across schools. The significance of the variance of these random slopes indicates whether the relationship between the predictor (competency at Year 6) and outcome (reading/numeracy score at Year 7) varied significantly according to school membership. These models also included estimates of a correlation between the random slopes and the intercept of the outcome variable. This tested whether the relationship between the predictor (Year 6 competency) and outcome (Year 7 academic achievement score) varied according to the schools’ average level of the outcome.

Supplementary Table B (below) displays the results of the random intercepts and coefficients model. For Self-Awareness models, significant variation in slope was observed, finding that the association between this social-emotional competency and academic achievement varied significantly between schools. A significant effect of slope correlation was observed in the Self-Awareness and numeracy model, indicating Self-Awareness had a stronger relationship with Year 7 numeracy in schools with higher average numeracy levels. This suggests that among high performing peers, students’ own confidence in their numeracy abilities has a strong influence on their performance, whereas the association between Self-Awareness and numeracy is weaker at lower performing schools. A trend effect (meeting the unadjusted threshold of *p* <.05) was observed for the slope correlation in the Self-Awareness and reading model and the Self-Management and numeracy model. No significant variations in slope were observed in the Self-Management models.

**Supplementary Table B.** Unstandardized results of Two-Level Structural Equation Models exploring the bivariate relationship between each social-emotional competency (Year 6) and reading and numeracy attainment (Year 7), accounting for covariates and permitting between-school variation in associations.

|  | **Reading** | | | **Numeracy** | | |  |
| --- | --- | --- | --- | --- | --- | --- | --- |
| **Parameter** | **B** | ***SE*** | ***t*** | **B** | ***SE*** | ***t*** | |
| *Self-Awareness* |  |  |  |  |  |  | |
| SEA1 Item Loading | 1.000 |  |  | 1.000 |  |  | |
| SEA2 Item Loading | 0.889 |  |  | 0.898 |  |  | |
| SEA3 Item Loading | 0.480 |  |  | 0.476 |  |  | |
| SEA4 Item Loading | 0.754 |  |  | 0.740 |  |  | |
| SEA Coefficient | 0.023 | (0.001) | 18.13*** | 0.036 | (0.001) | 25.72*** | |
| Sex Coefficient | -0.008 | (0.006) | -1.39 | -0.024 | (0.006) | -4.29*** | |
| PEd Coefficient | -0.057 | (0.009) | -6.18*** | -0.046 | (0.008) | -5.53*** | |
| Dis Coefficient | -0.053 | (0.017) | -3.09** | -0.096 | (0.013) | -7.20*** | |
| Yr5 Coefficient | 0.630 | (0.004) | 142.85*** | 0.713 | (0.005) | 131.37*** | |
| Slope Variance | 0.000 | (0.000) | 2.38* | 0.000 | (0.000) | 3.39** | |
| Slope Correlation | 3.741 | (1.825) | 2.05^ | 8.439 | (1.784) | 4.73*** | |
| *Self-Management* |  |  |  |  |  |  | |
| SM1 Item Loading | 1.000 |  |  | 1.000 |  |  | |
| SM2 Item Loading | 0.766 |  |  | 0.769 |  |  | |
| SM3 Item Loading | 0.388 |  |  | 0.392 |  |  | |
| SM Coefficient | 0.011 | (0.001) | 8.62*** | 0.019 | (0.001) | 12.99*** | |
| Sex Coefficient | 0.008 | (0.006) | 1.28 | -0.008 | (0.006) | -1.31 | |
| PEd Coefficient | -0.055 | (0.009) | -5.96*** | -0.045 | (0.009) | -5.25*** | |
| Dis Coefficient | -0.054 | (0.017) | -3.14** | -0.095 | (0.013) | -7.16*** | |
| Yr5 Coefficient | 0.654 | (0.004) | 157.24*** | 0.762 | (0.005) | 142.98*** | |
| Slope Variance | 0.000 | (0.000) | 0.98 | 0.000 | (0.000) | 1.77 | |
| Slope Correlation | -4.980 | (5.595) | -0.89 | 5.020 | (2.400) | 2.09^ | |

*Note.* *** *p*< .001; ** *p*< .01; * *p*< .034 (Bonferroni-adjusted significance threshold for 4 models and 0.730 correlation between reading and numeracy), ^ *p*< .050 (uncorrected threshold). SEA = Self-Awareness; SM = Self-Management.

**Table S6.** Results of the Two-Level Structural Equation Model (Model 1) exploring the relationship between both Self-Awareness and Self-Management (Year 6) and reading and numeracy attainment (Year 7).

|  | **Reading** | | | | **Numeracy** | | | |
| --- | --- | --- | --- | --- | --- | --- | --- | --- |
| **Parameter** | **β** | **B** | ***SE*** | ***t*** | **β** | **B** | ***SE*** | ***t*** |
| SEA1 Item Loading | 0.866 | 1.000 |  |  | 0.867 | 1.000 |  |  |
| SEA2 Item Loading | 0.846 | 0.914 |  |  | 0.847 | 0.918 |  |  |
| SEA3 Item Loading | 0.650 | 0.492 |  |  | 0.647 | 0.488 |  |  |
| SEA4 Item Loading | 0.772 | 0.700 |  |  | 0.772 | 0.699 |  |  |
| SM1 Item Loading | 0.854 | 1.000 |  |  | 0.857 | 1.000 |  |  |
| SM2 Item Loading | 0.762 | 0.717 |  |  | 0.762 | 0.707 |  |  |
| SM3 Item Loading | 0.597 | 0.454 |  |  | 0.593 | 0.443 |  |  |
| SEA SM Correlation | 0.351 | 0.998 |  |  | 0.351 | 1.013 |  |  |
| SEA Coefficient | 0.390 | 0.138 | (0.004) | 37.31*** | 0.506 | 0.178 | (0.004) | 41.66*** |
| SM Coefficient | 0.071 | 0.027 | (0.004) | 7.42*** | 0.002 | 0.008 | (0.003) | 2.42* |

*Note.* *** *p*< .001; ** *p*< .01; * *p*< .041 (Bonferroni-adjusted for 2 models and 0.730 correlation between reading and numeracy). SEA = Self-Awareness; SM = Self-Management. R^2^ values = 0.18 and 0.26 for reading and numeracy respectively.

**Table S7.** Results of the Two-Level Structural Equation Model (Model 2) exploring the relationship between both Self-Awareness and Self-Management (Year 6) and reading and numeracy attainment (Year 7), and accounting for covariates.

|  | **Reading** | | | | **Numeracy** | | | |
| --- | --- | --- | --- | --- | --- | --- | --- | --- |
| **Parameter** | **β** | **B** | ***SE*** | ***t*** | **β** | **B** | ***SE*** | ***t*** |
| SEA1 Item Loading | 0.845 | 1.000 |  |  | 0.840 | 1.000 |  |  |
| SEA2 Item Loading | 0.816 | 0.893 |  |  | 0.813 | 0.902 |  |  |
| SEA3 Item Loading | 0.601 | 0.476 |  |  | 0.597 | 0.481 |  |  |
| SEA4 Item Loading | 0.744 | 0.704 |  |  | 0.738 | 0.705 |  |  |
| SM1 Item Loading | 0.837 | 1.000 |  |  | 0.839 | 1.000 |  |  |
| SM2 Item Loading | 0.762 | 0.770 |  |  | 0.760 | 0.756 |  |  |
| SM3 Item Loading | 0.543 | 0.424 |  |  | 0.549 | 0.425 |  |  |
| SEA SM Correlation | 0.287 | 0.692 |  |  | 0.296 | 0.708 |  |  |
| SEA Coefficient | 0.097 | 0.041 | (0.002) | 18.86*** | 0.143 | 0.064 | (0.003) | 21.23*** |
| SM Coefficient | 0.021 | 0.009 | (0.002) | 3.88*** | 0.036 | 0.016 | (0.002) | 6.86*** |
| Sex Coefficient | -0.002 | -0.002 | (0.005) | -0.42 | -0.018 | -0.025 | (0.005) | -4.80*** |
| PEd Coefficient | -0.027 | -0.058 | (0.008) | -7.01*** | -0.022 | -0.048 | (0.008) | -6.09*** |
| Dis Coefficient | -0.016 | -0.061 | (0.012) | -4.91** | -0.028 | -0.105 | (0.014) | -7.58*** |
| Yr5 Coefficient | 0.796 | 0.659 | (0.003) | 204.90*** | 0.831 | 0.770 | (0.003) | 242.22*** |

*Note.* *** *p*< .001; ** *p*< .01; * *p*< .041 (Bonferroni-adjusted for 2 models and 0.730 correlation between reading and numeracy). SEA = Self-Awareness; SM = Self-Management; PEd = Parental Education; Dis = Disability; Yr5 = Year 5 NAPLAN score. R^2^ values = 0.66 and 0.73 for reading and numeracy respectively.

**Table S8.** Unstandardized results of Multi-Group Two-Level Structural Equation Models examining the relationship between each social-emotional competency (Year 6) and reading and numeracy attainment (Year 7) according to parents’ level of education, accounting for covariates.

|  | **Reading** | | | | | | | | **Numeracy** | | | | | | | |  |
| --- | --- | --- | --- | --- | --- | --- | --- | --- | --- | --- | --- | --- | --- | --- | --- | --- | --- |
|  | **Parental Education (> Year 12)** | | | | **Parental Education (< Year 12)** | | | | **Parental Education (> Year 12)** | | | | **Parental Education (< Year 12)** | | | | |
| **Parameter** | **β** | **B** | ***SE*** | ***t*** | **β** | **B** | ***SE*** | ***t*** | **β** | **B** | ***SE*** | ***t*** | **β** | **B** | ***SE*** | ***t*** | |
| *Self-Awareness* |  |  |  |  |  |  |  |  |  |  |  |  |  |  |  |  | |
| SEA Coefficient | 0.115 | 0.024 | 0.001 | 17.94*** | 0.089 | 0.017 | 0.003 | 5.08*** | 0.175 | 0.036 | 0.001 | 25.18*** | 0.162 | 0.032 | 0.003 | 10.69*** | |
| Wald test^a^ (*df*) | 3.56 (1) | | | | | | | | 1.21 (1) | | | | | | | | |
| Sex Coefficient | -0.005 | -0.007 | 0.007 | -1.09 | -0.012 | -0.015 | 0.017 | -0.88 | -0.019 | -0.024 | 0.006 | -3.99*** | -0.011 | -0.014 | 0.015 | -0.92 | |
| Dis Coefficient | -0.017 | -0.061 | 0.019 | -3.15** | -0.004 | -0.012 | 0.037 | -0.33 | -0.027 | -0.099 | 0.015 | -6.46*** | -0.028 | -0.081 | 0.030 | -2.65** | |
| Yr5 Coefficient | 0.784 | 0.631 | 0.005 | 139.29*** | 0.753 | 0.617 | 0.014 | 42.62*** | 0.810 | 0.715 | 0.005 | 132.02*** | 0.800 | 0.707 | 0.017 | 41.97*** | |
| *Self-Management* |  |  |  |  |  |  |  |  |  |  |  |  |  |  |  |  | |
| SM Coefficient | 0.049 | 0.011 | 0.001 | 8.22*** | 0.074 | 0.016 | 0.004 | 4.13*** | 0.076 | 0.017 | 0.001 | 13.29*** | 0.099 | 0.022 | 0.003 | 6.95*** | |
| Wald test^a^ *(df)* | 1.34 (1) | | | | | | | | 1.68 (1) | | | | | | | | |
| Sex Coefficient | 0.006 | 0.008 | 0.007 | 1.27 | 0.002 | 0.003 | 0.017 | 0.15 | -0.007 | -0.009 | 0.006 | -1.38 | 0.006 | 0.007 | 0.016 | 0.47 | |
| Dis Coefficient | -0.017 | -0.062 | 0.019 | -3.25** | -0.005 | -0.015 | 0.038 | -0.40 | -0.025 | -0.097 | 0.015 | -6.32*** | -0.029 | -0.087 | 0.030 | -2.88** | |
| Yr5 Coefficient | 0.797 | 0.657 | 0.004 | 155.16*** | 0.757 | 0.628 | 0.014 | 44.83*** | 0.829 | 0.765 | 0.005 | 145.78*** | 0.810 | 0.739 | 0.017 | 44.44*** | |

*Note.* *** *p*< .001; ** *p*< .010; * *p*< .034 (corrected threshold). ^a^ Wald test of the significance of the difference between the social-emotional coefficient parameters of children with higher and lower parental education; *df* = degrees of freedom; SEA = Self-Awareness; SM = Self-Management; Dis = Disability; Yr5 = Year 5 reading or numeracy score.
